# Effects of acidic water in combination with aluminum on swimming behavior and survival of yolk-sac larval in Goldfish (*Carassius auratus gibelio*)

**DOI:** 10.1186/2193-1801-2-190

**Published:** 2013-04-27

**Authors:** Vahid Taghizadeh, Mohammad Reza Imanpoor, Mahboubeh Hosseinzadeh, Hajar Azarin

**Affiliations:** Department of Fisheries, Gorgan University of Agricultural Sciences and Natural Resources, Gorgan, Iran

**Keywords:** *Carassius auratus gibelio*, acidic waters, aluminum, swimming activity, heartbeat

## Abstract

Yolk-sac fry of Goldfish (*Carassius auratus gibelio*) were exposed to various concentrations of Al and pH for 3, 7 and 10 days. In this study number of dead fish, total length of larvae, yolk sac length, swimming activity, the number of gill cover movements and the number of heart beat was measured. The lowest and highest number of gill cover movements belong to the waters containing 50 mg L-1 of aluminum, pH 5.25 and acidic waters with pH 6.7 respectively. There was no significant difference in the number of heartbeat between control group and larvae exposure to acidic waters with pH 6.7 and waters containing 150 mgL-1 of aluminum and the highest number of heartbeat belong to these larvae than other experiment doses. The larval of Goldfish in the all experiment doses lost their swimming ability after 3 days. The highest number of swimming larvae and also lowest number of non-swimming larvae significantly belong to control group compared to other experiment doses (p < 0.05). The larval in the control group only were maintained their swimming activity after 7 and 10 days and in the all experiment treatments lost their swimming ability. There was significant difference in the number of dead larvae and the number of non-swimming larvae between control group and larvae exposure to acidic waters and waters containing 300 mgL-1 of aluminum and pH 4.75 (p < 0.05) and lowest number of non-swimming larvae was observed in the control group. Also the highest of yolk sac length belong to the waters containing 600 mgL-1 of aluminum and pH 4.75. The larvae of Goldfish were not survived in the waters containing 300 and 600 mgL-1 of aluminum and waters with low acidity after 7 days.

**Electronic supplementary material:**

The online version of this article (doi:10.1186/2193-1801-2-190) contains supplementary material, which is available to authorized users.

## Introduction

Fish mortality in acidic waters is determined by multiple factors, including pH, inorganic monomeric (exchangeable) Al and Ca^+2^ among the most important. Aluminum is a common element in the Earth crust. The solubility of aluminum in water increases as pH decreases (Smith and Haines, 1995).

The toxic effect of aluminum on fish varies with both fish species and life stages. Yolk-sac fry are more sensitive than eggs, but following yolk-sac absorption, sensitivity increases yet further. It is generally believed that sensitivity to aluminum toxicity at low pH increases with age (Catla 2002). The threshold pH values with respect to survival and swimming activity (lowest pH for survival and safe pH) were, dependingon the Al concentration.

Fish reproduction is considered sensitive to water acidification (Peterson et al., 1982), as reflected in fish population decline in lakes where, overall, few adult fish mortalities have been reported (Rask and Tuunainen, 1990).

In yolk-sac fry, the routine metabolic rate may, in contrast, be reduced as acidity decreases their swimming activity as documented mainly in salmonoid fishes (Sayer et al., 1993a), but also in such species as small Mouth bass (*Micropterus dolomieu*) (Kane and Rabeni, 1987), Whitefish, Pike-perch, Roach and Pike (Vuorinen et al. 1993).

Aluminum is one of the important factors in the toxicity of acidified waters to freshwater fish species because low pH and high concentrations of Al have been of particular concern in affected waters. Al toxicity is mostly associated with the acidification because its solubility increases exactly below pH 4.0 and it becomes the most important factor responsible for fish kills in acidified water bodies (Mani Sharma, 2003).

Acidic water and Al interferes with the ionbalance of fish by increasing ion efflux and by inhibiting the uptake of essential ions from the environment. Acidic water and Al also Ca use internal hypoxia which is seen, for example, as increased ventilation rate and may result from the precipitation or polymerization of Al onto the gills. Increased mucus secretion, and thickening or more striking changes in the bronchial epithelium may also occur. Innewly-hatched fry the gas exchange is mainly cutaneous and the skin evidently functions in ion-exchange also. Aluminum in acidic water clearly augments both the decrease of activity and retardation of yolk absorption and consequently the development and growth of yolk-sac fry, seen especially in species with a large yolk-sac and a long yolk-sac phase (Keinanen et al., 2000).

Sub lethal acidified conditions may increase the basal metabolic rate because more energy is needed to maintain the ion balance (McCormick et al. 1989; Leino et al., 1990; Wilson et al., 1994). Research into the susceptibility of early life phases of fish to acidity has mainly focused on salmonoid species (Sayer et al. 1993b).

However, little is known concerning the basis for the species differences in tolerant of acidified waters and aluminum. To accomplish this, we exposed yolk-sac fry of Goldfish to realistic conditions of acidity alone, and in combination with aluminum. Thus, the main goal of the present study was to find the physiological basis for the differences in sensitivity to acidic water and Al in newly-hatched yolk-sac fry of Goldfish. This was investigated by exposing newly-hatched yolk-sac fry to various combinations of pH values and Al concentrations, and measuring parameters such as yolk-sac length, swimming activity, the number of gill cover movements and the number of heartbeat in yolk-sac fry of Goldfish (*Carassius auratus gibelio*).

## Materials and methods

Three experiments were performed in which newly-hatched yolk-sac fry were exposed to different combinations of acidic water and Al in the first experiment (experiment 1) mortality, swimming activity, movements of the operculum, and in second experiment (experiment 2) mortality, swimming activity and yolk-sac length and in third experiment (experiment 3) mortality, swimming activity, total length, yolk-sac length, heartbeats and movements of the operculum were measured.

### Fish experiment

In this study we used from Goldfish larvae. Two injections of Ova prim were used to simulate final maturation and artificial propagation in the female Goldfish broodstock. In females the first injection (5% of total dose) was made at 10 pm and second (95% of total dose) 24 h later at 8 am hours. And males were injected as time as the second injection in females. And 24–48 h after final injection Oocytes and milt were stripped and mixed together using a light feather. After adhesion removal (2 ppt NaCl solution), the fertilized eggs were transferred into glass aquarium supplied with gentle aeration. After 48 h the eggs were hatched (at 23°C). Newly-hatched larvae with 7 ± 1mm length and 32.33 ± 1.15 mm yolk-sac length were used in the tests.

### Preparation of test solution

The test waters for experiments were prepared from urban water, after chlorinization, which also served as the control. The test water for experiments 1, 2 and 3 were made by dissolving aluminum (Al_2_ (SO_4_))_3_ into deionized water. The pH of test solutions was checked daily and fixed by adding Tris and Tris Hcl. The water was aerated after each acid addition. A series of test solutions was made for each experiment and was used throughout the test.

### Experimental protocol

Exposures were conduct in glass jars, the water volume of which was 300 ml. each experiment was carried out as three replicates using 30 yolk-sac fry. Temperature was maintained at 18 ± 2°C. Approximately 80% of the test ware was renewed at least every second day. Fry were not fed; they resorbed their yolk during the experiments.

### Experiment 1

Mortality, swimming activity, movements of the operculum were recorded. Goldfish fry were exposed for 3 days to acid water at pH 5.00, 5.25, 5.75, and 6.7, and to Al in concentrations of 0, 50, 150, and 250 mg L^-1^. The water temperature was 18 ± 2°C (mean ± SD of daily measurements). Dead fry were removed and numbers of swimming and non-swimming fry were registered daily. The movements of the operculum of fry were monitored under the microscope. Before the estimations, the fry were allowed to calm down for at least 30 s after putting them onto a Petri dish the time for 30 movements of the operculum was measured three times in ten fry in each group.

### Experiment 2

Mortality, swimming activity and yolk-sac length were recorded. Goldfish fry were exposed for 7 days to acid water at pH 4, 4.25, 4.75 and 5, and to Al in concentration of 0, 300 and 600 mg L^-1^. The water temperature was 18 ± 2°C (mean ± SD of daily measurements).

### Experiment 3

Mortality, swimming activity, total length, heartbeats and movements of the operculum were measured. Goldfish fry were exposed for 10 days to acid water at pH 6.50, and to Al in concentrations of 0, 50, and 100 mg L^-1^. The water temperature was 18 ± 2°C (mean ± SD of daily measurements). The movements of the operculum of fry were monitored under the microscope. Before the estimations, the fry were allowed to calm down for at least 30 s after putting them onto a Petri dish the time for 30 movements of the operculum was measured three times in ten fry in each group. Similarly, eight fry from each group were used to measure the time needed for 30 heartbeats and at least three repetitive measurements were taken.

### Data analyze

Two-way ANOVA was applied to test the effects of Al and pH on length, movements of operculum, heartbets, and summing activity. The differences between the means were tested by Duncan test at the 95% confidence level.

## Results

After 42h, 100% mortality was observed in pH 5. 25 at AL 0, 150 mg L^-1^ and 250, pH 5.75 at Al 50, and 150 mg L^-1^ in treatment 1, and in pH 4 at AL 0, 300, 600 mg L^-1^, pH 4.25 at Al 300 and 600 mg L^-1^, pH 4.5 at Al 300 and 600 mg L^-1^ and in pH 5 at Al 300 and 600 mg L^-1^ in treatment 2.

The results of Analyze-Variance and the means comparison of evaluated parameters of yolk-sac fry of Goldfish after exposure to different concentrations of Al and pH for 3 days are shown in Table 1.

**Table 1:** **The means comparison of evaluated parameters of yolk-sac fry of Goldfish after exposure to different concentrations of Al (mg L** ^**-1**^ **) and pH for 3 days**

Al- pH	Dead (Number)	Gill cover movements (Number)	Non-swimming (Number)	Swimming (Number)
Control	^d^0 ± 0	^a^1.52 ± 19.33	^d^0 ± 0	^a^0 ± 30
0-5.75	^c^9.60 ± 8.66	^a^4.72 ± 19.66	^b^9.6 ± 21.33	^c^0 ± 0
0-6.7	^d^0 ± 0	^a^2 ± 17	^a^0 ± 30	^c^0 ± 0
50-0	^d^1.5 ± 1.33	^b^2 ± 17	^a^1.52 ± 28.66	^c^0 ± 0
50-5.25	^d^0 ± 0	^b^4.9 ± 16.66	^a^0 ± 30	^c^0 ± 0
50-6.7	^d^0 ± 0	^a^2.51 ± 19.33	^a^0 ± 30	^c^0 ± 0
150-0	^d^0 ± 0	^a^5.56 ± 19	^a^0 ± 30	^c^0 ± 0
150-5.75	^b^2 ± 24	^b^2.6 ± 16	^c^2 ± 6	^c^0 ± 0
150-6.7	^d^0 ± 0	^a^6.5 ± 18.33	^a^0 ± 30	^c^0 ± 0
250-5.75	^b^5 ± 20	^a^7.3 ± 23.66	^c^5 ± 10	^c^0 ± 0
250-6.7	^d^0 ± 0	^a^10.9 ± 26.33	^a^1.7 ± 29	^b^1.7 ± 1

Different letters denote a significant difference at the same column (P < 0.05).

The larvae of Goldfish in the all experiment doses lost their swimming ability after 3 days and all larvae were non-swimming in acidic waters with pH 6.7 and waters containing 50 mg L^-1^ aluminum and pH 5.25 and pH 6.7, waters containing 50 mg L^-1^ of aluminum and pH 6.7 and also waters containing 250 mg L^-1^ aluminum and pH 6.7 (Table 1). The highest number of non-swimming larvae was observed in the water containing 50 mg L^-1^ aluminum and pH 5.25 and pH 6.7 and also waters with 150 mg L^-1^ aluminum and pH 6.7. The highest number of live and swimming larvae and also lowest number of non-swimming larvae significantly belong to control group compared to other experiment doses (P < 0.05). There was no significant difference in the number of gill cover movements between control group and larvae exposure to acidic waters with pH 5.57 and pH 6.7, waters containing 50 mg L^-1^ of aluminum and pH 6.7 and also waters containing 150 mg L^-1^ of aluminum and pH 6.7 (P < 0.05). Also, the highest number of gill cover movements was observed in these groups (Table 1).

Yolk-sac fry of Goldfish were exposed to concentrations of 0, 300 and 600 mgL^-1^ of aluminum and pH 4, 4.25, 4.5, 4.75 and 5 for 10 days but after 7 days, only control group and larvae were exposed to acidic waters with pH 4.25, 4.5, 4.75 and 5 and waters containing 300 mg L^-1^ of aluminum and pH 4.57 and containing 600 mg L^-1^ of aluminum and pH 4.57 remained alive. The results of the means comparison of larvae were placed at doses above are shown in Table 2.

**Table 2:** **The means comparison of evaluated parameters of yolk-sac fry of Goldfish after exposure to different concentrations of Al and pH for 7 days**

Parameters	Control	4.25-0	4.5-0	4.75-0	5-0	4.75-300	4.75-600
Dead (Number)	^b^0 ± 0	^a^8.5 ± 17.66	^a^5.5 ± 18.66	^a^1.5 ± 21.66	^b^1 ± 3	^a^1.5 ± 24.66	^a^2.5 ± 20.66
Swimmer (Number)	^a^0 ± 30	^b^0 ± 0	^b^0 ± 0	^b^0 ± 0	^b^0 ± 0	^b^0 ± 0	^b^0 ± 0
Non-Swimming (Number)	^c^0 ± 0	8.5 ^b^ ± 12.33	5.5 ^b^ ±11.33	1.5 ^b^ ±8.33	1 ^a^ ±27	1.5 ^b^ ±5.33	2.51 ^b^ ±9.33
Yolk-sac length (mm)	^d^0.02 ± 0.1	^b^0.3 ± 1.19	^b^0.35 ± 1.10	^c^0.04 ± 0.62	^c^0.03 ± 0.48	^b^0.19 ± 1.17	^a^0.23 ± 1.93

Different letters denote a significant difference at the same row (P < 0.05).

As shown in Table 2, the larvae in the control group only were maintained their swimming activity after 7 days and in the all experiment treatments lost their swimming ability. There was significant difference in the number of dead larvae and the number of non-swimming larvae between control group and larvae exposed to acidic waters and waters containing 300 mg L^-1^ of aluminum and pH 4.75 (p < 0.05) and lowest number of non-swimming larvae was observed in the control group. Also the highest of yolk-sac length belong to the waters containing 600 mg L^-1^ of aluminum and pH 4.75 and then highest of yolk-sac length belong to waters with pH 4.25 and pH 4.5 and also waters containing 300 mg L^-1^ of aluminum and pH 4.75. There was significant difference among control group and acidic waters and waters containing aluminum (Table 2).

larval were exposed in acidic waters with pH 6.5 and waters containing 50 and 100 mgL^-1^ of aluminum for the 10 days that results of means comparison this experimental doses are given in Table 3.

**Table 3:** **The means comparison of evaluated parameters of yolk-sac fry of Goldfish after exposure to 50 and 100 mgL** ^**-1**^ **of aluminum and acidic waters with pH 6.5 for 10 days**

Parameters	Control	6.5-0	6.5-50	6.5-100
Dead	0 ± 0 ^c^	^a^1.52 ± 18.33	^b^5.03 ± 10.66	^b^1.73 ± 11
Swimming	0 ± 30 ^a^	^c^0 ± 0	^b^8.08 ± 15.33	^c^0 ± 0
Non-swimmer	^c^0 ± 0	^b^1.52 ± 11.66	^b^6 ± 6	^a^1.73 ± 19
Total length (mm)	^a^0.57 ± 79.66	^b^2.51 ± 67.66	^a^0.57 ± 77.33	^a^1.15 ± 78.33
Gill cover movements	^a^1.52 ± 20.33	^b^1.52 ± 12.33	^b^1.52 ± 11.66	^b^1 ± 10
Heartbeat	^a^2 ± 42	^a^1 ± 40	^b^1.52 ± 35.33	^a^1.73 ± 41

Different letters denote a significant difference at the same row (P < 0.05).

As shown in Table 3, There was no significant difference between groups that exposed to waters containing 50 and 100 mgL^-1^ of aluminum and there was significant difference in number of dead larvae and number of swimmer larvae and also non swimmer larvae between control group and larvae exposed to waters containing 50 and 100 mgL^-1^ of aluminum and pH 6.5. The highest number of gill cover movements was observed in control group (P < 0.05). The lowest number of heartbeat significantly belong to larvae that were exposed 50 mgL^-1^ of aluminum and pH 6.5 than other experiment doses (P < 0.05) (Table 3).

## Discussion

### Mortality

Exposure of Goldfish to lower pH levels <5.75 reduced survival compared to pH levels of 6–7. In this study, high mortality occurred around pH 4–5.75. The highest mortality was observed in pH 5.75 after 3 days; this could be because of the disturbance of ion balance. Similarly, Howells et al. (1990) and Keinanen et al. (2000) concluded that the cause of death in aluminum exposed fish was predominantly the disturbance of ion balance. Because, acidic water and Al interferes with ion balance of fish by increasing ion efflux and by inhibiting the uptake of essential ion from the environment.

Several authors have demonstrated the influence of pH 4.5- 5.6 on aquaculture fish in a wide range of hardness variation. Jezierska and Witeska (1995) observed total mortality in common carp larvae at pH 5.5. In generally, most teleost exposed to acidic or alkaline waters showed a higher survival than in soft waters (Parra and Baldisserotto 2007).

After 7 days, the highest mortality was observed in pH 4–4.75 And Al 300 and 600 mgl^-1^. Some researchers showed that high concentration of Al in combination with low pH have been shown to cause mortality of freshwater fish in both field and laboratory studies (Atland and Barlaup, 1995). Al toxicity depends on the species of Al present, which is largely dependent on pH (Burrows 1997). Therefore, in the present study, it was found that a combination of low pH and Al is toxic to larval.

It has been suggested that the embryonic and larval fish stages are most sensitive to pH changes (Heydarnejad, 2012). Heydarnejad (2012) stated that larval of common carp grew and survived best when exposed to a water pH of 7.5-8.

### Larval development

In the present study exposed larval have more yolk-sac remaining as compared with control group. Indeed, absorption of yolk-sac fry in acidic water was delayed. Consequently, decreasing of absorption of yolk-sac leading to reduced growth. Similarly, Catla (2002) stated that a sub-lethal exposure at an early age retards larval development and thus exposed animals have more yolk-sac remaining as compared with control animals.

Increasing of the retarded absorption of yolk and, as a consequence, retarded growth of yolk-sac fry by Al in acidic water was equally evident in some species including pike (Keinanen et al. 2000).

Decreasing of swimming in newly-hatched Goldfish in all of the concentration of Al after 7 and 10 days, indicate that Goldfish larval is sensitive to low pH and acidic waters. Similarly results were obtained by Vuorinen et al. (1993) and Keinanen et al. (2000). Al in acidic water clearly augments both the decrease of activity and retardation of yolk absorption and consequently the development and growth of yolk-sac fry (Vuorinen et al. 1993).

The present study shows that the potential for routine swimming behavior to recover during continued exposure is dose-dependent. It was the respiratory stress caused by exposure to Al which resulted in the shutdown of routine swimming behavior (Alin and Wilson 2000). In yolk- sac fry, the routine metabolic rate may, in contrast, be reduced as acidity decreases their swimming activity as documented mainly in salmonoid fishes (Sayer et al. 1993a. Sayer et al. 1993b).

Allin and Wilson (1999) recently provided evidence for a loading influence of Al and low pH on metabolism by showing that juvenile rainbow trout reduce their swimming activity in order to maintain their routine metabolic rate at control levels when exposed to acidic water and Al.

In the present study, the movement of operculum decreased in acidic water at high Al concentration. Also, heart rate was lower in low pH and Al. similarly,Keinanen et al. (2000) stated that the ventilation rate and specific O_2_ consumption rate of Pike decreased most in acidic water at high Al concentrations. Also, Keinanen et al. (2000) observed that heart rate of Pike yolk-sac fry tendency toward a lower in more acidic and Al containing water.

## Conclusion

Goldfish larval can be tolerate wide range of water pH Higher survival was verified at pH between 5.75 and 6.7. Generally, low pH in combination with high Al is more toxic to larval Goldfish. This results showed that Goldfish larval is sensitive to low pH and acidic waters, and in acidic water swimming activity and absorption of yolk-sac is decreased.

## Authors’ original submitted files for images

Below are the links to the authors’ original submitted files for images. Authors’ original file for figure 1
