# Supplementary material for: Effects of acidic water in combination with aluminum on swimming behavior and survival of yolk-sac larval in Goldfish (Carassius auratus gibelio)
Source: Springerplus. 2013 Apr 27;2(1):190. doi: 10.1186/2193-1801-2-190 (PMC3650235; doi:10.1186/2193-1801-2-190)
Supplement: Supplementary file 1 — Authors’ original file for figure 1 [file 40064_2013_252_MOESM1_ESM.docx]

Table 1. The means comparison of evaluated parameters of yolk-sac fry of Goldfish after exposure

to different concentrations of Al (mg L^-1^) and pH for 3 days

| Al- pH | Dead  (Number) | Gill cover movements  (Number) | Non-swimming  (Number) | Swimming  (Number) |
| --- | --- | --- | --- | --- |
| Control | ^d^0±0 | ^a^1.52±19.33 | ^d^0±0 | ^a^0±30 |
| 0-5.75 | ^c^9.60± 8.66 | ^a^4.72±19.66 | ^b^9.6±21.33 | ^c^0±0 |
| 0-6.7 | ^d^0± 0 | ^a^2±17 | ^a^0±30 | ^c^0±0 |
| 50-0 | ^d^1.5± 1.33 | ^b^2±17 | ^a^1.52±28.66 | ^c^0±0 |
| 50-5.25 | ^d^0± 0 | ^b^4.9±16.66 | ^a^0±30 | ^c^0±0 |
| 50-6.7 | ^d^0± 0 | ^a^2.51±19.33 | ^a^0±30 | ^c^0±0 |
| 150-0 | ^d^0± 0 | ^a^5.56±19 | ^a^0±30 | ^c^0±0 |
| 150-5.75 | ^b^2±24 | ^b^2.6±16 | ^c^2±6 | ^c^0±0 |
| 150-6.7 | ^d^0±0 | ^a^6.5±18.33 | ^a^0±30 | ^c^0±0 |
| 250-5.75 | ^b^5±20 | ^a^7.3±23.66 | ^c^5±10 | ^c^0±0 |
| 250-6.7 | ^d^0±0 | ^a^10.9±26.33 | ^a^1.7±29 | ^b^1.7±1 |

Different letters denote a significant difference at the same column (P<0.05)

Table 2. The means comparison of evaluated parameters of yolk-sac fry of Goldfish after exposure

to different concentrations of Al and pH for 7 days

| Parameters | Control | 4.25-0 | 4.5-0 | 4.75-0 | 5-0 | 4.75-300 | 4.75-600 |
| --- | --- | --- | --- | --- | --- | --- | --- |
| Dead (Number) | ^b^0±0 | ^a^8.5±17.66 | ^a^5.5±18.66 | ^a^1.5±21.66 | ^b^1±3 | ^a^1.5±24.66 | ^a^2.5±20.66 |
| Swimmer (Number) | ^a^0±30 | ^b^0±0 | ^b^0±0 | ^b^0±0 | ^b^0±0 | ^b^0±0 | ^b^0±0 |
| Non-Swimming (Number) | ^c^0±0 | 8.5 ^b^±12.33 | 5.5 ^b^ ±11.33 | 1.5 ^b^ ±8.33 | 1 ^a^ ±27 | 1.5 ^b^ ±5.33 | 2.51 ^b^ ±9.33 |
| Yolk-sac length (mm) | ^d^0.02±0.1 | ^b^0.3±1.19 | ^b^0.35±1.10 | ^c^0.04±0.62 | ^c^0.03±0.48 | ^b^0.19±1.17 | ^a^0.23±1.93 |

Different letters denote a significant difference at the same row (P<0.05).

Table 3: The means comparison of evaluated parameters of yolk-sac fry of Goldfish after exposure to 50 and 100 mgL^-1^ of aluminum and acidic waters with pH 6.5 for 10 days

| Parameters | Control | 6.5-0 | 6.5-50 | 6.5-100 |
| --- | --- | --- | --- | --- |
| Dead | 0± 0 ^c^ | ^a^1.52± 18.33 | ^b^5.03± 10.66 | ^b^1.73± 11 |
| Swimming | 0± 30 ^a^ | ^c^0± 0 | ^b^8.08± 15.33 | ^c^0± 0 |
| Non-swimmer | ^c^0± 0 | ^b^1.52± 11.66 | ^b^6± 6 | ^a^1.73± 19 |
| Total length (mm) | ^a^0.57± 79.66 | ^b^2.51± 67.66 | ^a^0.57± 77.33 | ^a^1.15± 78.33 |
| Gill cover movements | ^a^1.52± 20.33 | ^b^1.52± 12.33 | ^b^1.52±11.66 | ^b^1± 10 |
| Heartbeat | ^a^2± 42 | ^a^1± 40 | ^b^1.52± 35.33 | ^a^1.73± 41 |

Different letters denote a significant difference at the same row (P<0.05)
